# Pharmacological doses of niacin stimulate the expression of genes involved in carnitine uptake and biosynthesis and improve the carnitine status of obese Zucker rats

**DOI:** 10.1186/2050-6511-15-37

**Published:** 2014-07-09

**Authors:** Aline Couturier, Robert Ringseis, Erika Most, Klaus Eder

**Affiliations:** 1 Institute of Animal Nutrition and Nutrition Physiology, Justus-Liebig-University Giessen, Heinrich-Buff-Ring 26-32, 35390 Giessen, Germany

**Keywords:** Niacin, Carnitine synthesis, Peroxisome proliferator-activated receptor α, Zucker rats

## Abstract

**Background:**

Activation of peroxisome proliferator-activated receptor (PPAR)α and PPARδ causes an elevation of tissue carnitine concentrations through induction of genes involved in carnitine uptake [novel organic cation transporter 2, (OCTN2)], and carnitine biosynthesis [γ-butyrobetaine dioxygenase (BBD), 4-*N*-trimethyl-aminobutyraldehyde dehydrogenase (TMABA-DH)]. Recent studies showed that administration of the plasma lipid-lowering drug niacin causes activation of PPARα and/or PPARδ in tissues of obese Zucker rats, which have a compromised carnitine status and an impaired fatty acid oxidation capacity. Thus, we hypothesized that niacin administration to obese Zucker rats is also able to improve the diminished carnitine status of obese Zucker rats through PPAR-mediated stimulation of genes involved in carnitine uptake and biosynthesis.

**Methods:**

To test this hypothesis, we used plasma, muscle and liver samples from a recent experiment with obese Zucker rats, which were fed either a niacin-adequate diet (30 mg niacin/kg diet) or a diet with a pharmacological niacin dose (780 mg niacin/kg diet), and determined concentrations of carnitine in tissues and mRNA and protein levels of genes critical for carnitine homeostasis (OCTN2, BBD, TMABA-DH). Statistical data analysis of all data was done by one-way ANOVA, and Fisher’s multiple range test.

**Results:**

Rats of the obese niacin group had higher concentrations of total carnitine in plasma, skeletal muscle and liver, higher mRNA and protein levels of OCTN2, BBD, and TMABA-DH in the liver and higher mRNA and protein levels of OCTN2 in skeletal muscle than those of the obese control group (P < 0.05), whereas rats of the obese control group had lower concentrations of total carnitine in plasma and skeletal muscle than lean rats (P < 0.05).

**Conclusion:**

The results show for the first time that niacin administration stimulates the expression of genes involved in carnitine uptake and biosynthesis and improves the diminished carnitine status of obese Zucker rats. We assume that the induction of genes involved in carnitine uptake and biosynthesis by niacin administration is mediated by PPAR-activation.

## Background

Niacin (nicotinic acid), a water-soluble vitamin of the B-complex, is involved in many metabolic pathways as a precursor of the redox coenzymes nicotinate adenine dinucleotide (NAD) and NAD phosphate [1]. Besides, niacin has potent plasma lipid-lowering effects and has long been used for clinical therapy of dislipidemia, particularly hypertriglyceridemia, due to the fact that niacin leads to decreased levels of triglycerides (TAG), very low-density lipoproteins (VLDL) and LDL in plasma [1-5]. Even though it has been established that niacin inhibits lipolysis in adipocytes and thereby reduces the supply of non-esterified fatty acids (NEFA) for hepatic TAG synthesis, this effect can only insufficiently explain the lipid-lowering effect because blood NEFA levels often rebound during long-term niacin treatment while the lipid-lowering effect remains [1,6].

With regard to other effects of niacin, which may contribute to its lipid-lowering efficacy, it is noteworthy that acute administration of niacin causes an increase of the mRNA levels of peroxisome proliferator-activated receptor (PPAR)α, PPARδ and the PPAR co-activator PGC-1α in tissues of male healthy subjects [7]. PPARα and PPARδ are ligand-dependent transcription factors which act as important metabolic regulators, especially in fatty acid catabolism, and are therefore abundantly expressed in tissues with high rates of fatty acid oxidation such as liver and skeletal muscle [8]. Endogenous ligand-activation is mediated by NEFA and, physiologically occurs during fasting when NEFA are released from adipose tissue und taken up into liver, muscle and other tissues [9,10]. Transcriptional regulation of genes by PPARs is mediated by binding of activated PPAR/retinoid X receptor heterodimers to specific DNA sequences, called peroxisome proliferator response elements, present in and around the promoter of target genes [8,11,12]. Typical PPARα and PPARδ target genes are involved in all aspects of fatty acid catabolism, such as fatty acid uptake, intracellular fatty acid transport, mitochondrial fatty acid import, and peroxisomal and mitochondrial fatty acid oxidation [8,13]. In addition to its function in fatty acid catabolism, it has been recently established that PPARα and PPARδ are transcriptional regulators of genes involved in carnitine uptake, like novel organic cation transporter 2 (OCTN2, encoded by SLC22A5) [12,14], and/or carnitine biosynthesis, such as γ-butyrobetaine dioxygenase (BBD, encoded by BBOX1) and 4-*N*-trimethyl-aminobutyraldehyde dehydrogenase (TMABA-DH, encoded by ALDH9) [15,16]. This explains why physiological or pharmacological PPARα and PPARδ activation causes an elevation of carnitine concentrations in tissues because both, carnitine uptake and biosynthesis is stimulated [14,17,18]. Transcriptional regulation of genes involved in carnitine homeostasis by PPARα and PPARδ is in line with their fundamental role for fatty acid catabolism because oxidation of fatty acids requires their import into the mitochondrial matrix, which is dependent on carnitine.

Interestingly, we have recently observed that administration of a pharmacological dose of niacin to obese Zucker rats causes elevated mRNA levels of a large set of PPARα and PPARδ target genes involved in fatty acid catabolism in skeletal muscle, and markedly lowers plasma lipid levels [19]. This indicates that niacin administration causes activation of PPARα and/or PPARδ in tissues of obese Zucker rats and that the niacin-induced improvement of fatty acid oxidation capacity of skeletal muscle contributes to the lipid-lowering effect of niacin. Since PPARα and PPARδ activation stimulates carnitine uptake and biosynthesis, it is likely that niacin enhances tissue carnitine concentrations, which also contributes to the improvement of mitochondrial fatty acid utilization. In light of this, we hypothesized that niacin administration causes a stimulation of the expression of genes involved in carnitine uptake and biosynthesis and thereby increases tissue carnitine concentrations. Considering that genetic or diet-induced obesity in rodents was found to result in a compromised carnitine status due to a reduced capacity of the body to synthesize and take up carnitine [20-22], it may be expected that such an effect of niacin is particularly useful in obesity. To test our hypothesis, we therefore used plasma, muscle and liver samples from a recent experiment with obese Zucker rats, an established genetic model of human obesity, and determined concentrations of carnitine and mRNA and protein levels of genes critical for carnitine homeostasis in liver and skeletal muscle [19].

## Methods

### Animals and diet

The obese (fa/fa) Zucker rat (Crl:ZUC-*Lepr*^*fa*^; Charles River, France) was used as animal model, which is a widely used genetic model of obesity, metabolic syndrome and diabetes. For this study, we used plasma, muscle and liver samples from an animal experiment performed recently by our group [19], in which twelve, 8- to 10-wk old male obese Zucker rats were randomly divided into two groups of each 10 rats. In addition, 10 male heterozygote lean (fa/+) Zucker rats were used. The rats received two different semi-purified diets which were composed according to the recommendations of the American Institute of Nutrition (AIN)-93G [23]. The first diet containing 30 mg supplemented niacin per kg diet, which was sufficient to cover the niacin requirement, was fed to the lean group and the obese control group, whereas the second diet containing 780 mg supplemented niacin (Lonza, Basel, Switzerland) per kg diet was fed to the obese niacin group. The lean group served as a “healthy” reference group in order to demonstrate the effect of the obese phenotype on carnitine homeostasis. A lean niacin group was not included in this experiment, because niacin at pharmacological doses does not exert a lipid-lowering action in healthy subjects with normal blood lipid levels and was not expected to cause an alteration of carnitine homeostasis. The diets were fed *ad libitum* and water was available *ad libitum* for a period of 28 days. Further details regarding diet composition, animal keeping and sample collection are shown in our previous publication [19]. In Accordance with Article 4 par. 3 of the German Animal Welfare Law all animals were humanely killed for scientific purpose approved by the Animal Welfare Officer of the Justus-Liebig-University, JLU No. 450_AZ.

### Determination of plasma concentrations of nicotinic acid, nicotinamide (NAM), and nicotinuric acid (NUA)

The concentrations of nicotinic acid, NAM and NUA in plasma were determined by LC-MS/MS according to the method from Liu et al. [24] with slight modifications which have been reported recently [25].

### Determination of concentrations of total carnitine and carnitine precursors in plasma, muscle and liver

Tandem mass spectrometry was used for determining the concentrations of free carnitine, acetyl-carnitine, and carnitine precursors [6-N-trimethyllysine (TML), butyrobetaine (BB)] in plasma, *M. gastrocnemius* and liver according the method of Hirche et al. [26]. Total carnitine was calculated as the sum of free carnitine and acetyl-carnitine. Deuterated carnitine-d3 (Cambridge Isotype Laboratories, Andover, MA, USA) was used as internal standard.

### RNA isolation and qPCR (quantitative realtime RT-PCR) analysis

Total RNA was isolated from 20 mg skeletal muscle and liver tissue using Trizol™ reagent (Invitrogen, Karlsruhe, Germany) according to the manufacturer’s protocol. RNA isolation, cDNA synthesis and qPCR were performed as recently described [27]. The three most stable out of six tested potential reference genes were *CANX, TOP1, YWHAZ* in liver and *RPL13, TOP1, YWHAZ* in *M. gastrocnemius*. Data on qPCR performance for each gene measured in liver are shown in Table 1.

**Table 1:** **Characteristics and performance data of the primers used for reference gene-stability measure *M *** **and qPCR**

**Gene symbol**	**Primer sequence (forward, reverse; from 5’ to 3’)**	**NCBI GeneBank**	**Product size (bp)**	**Skeletal muscle**			**Liver**		
				**Slope**	**R**^**2#**^	Efficiency*	**Slope**	**R**^**2#**^	Efficiency*
ACADL	AAGGATTTATTAAGGGCAAGAAGC	NM_012819.1	380 bp	-4.45	1.000	1.68	-3.54	0.999	1.92
	GGAAGCGGAGGCGGAGTC								
ACADM	CAAGAGAGCCTGGGAACTTG	NM_016986.2	154 bp	-3.29	0.998	2.01	-3.29	0.998	2.01
	CCCCAAAGAATTTGCTTCAA								
ACADS	ACATCTCTTCCCCACATCGC	NM_022512.2	204 bp	-2.98	0.999	2.16	-3.55	0.999	1.91
	CCGAACTTCAGGATGGGTCC								
ACOX1	CTGGGCTGAAGGCTTTTACT	NM_017340.2	172 bp	-3.60	0.997	1.90	-3.97	0.999	1.79
	GCTGTCTGCAGCATCATAAC								
ALDH9	TTGAGCGGCTGCGACACGAC	NM_022273.2	82 bp		-		-3.42	0.999	1.96
	TGACCTCGCTCCTCCGCGTA								
ATP5B	GCACCGTCAGAACTATTGCT	NM_134364	203 bp	-3.58	0.998	1.90	-3.33	0.999	2.00
	GAATTCAGGAGCCTCAGCAT								
BBOX1	GGATGGGGCTCGCTTGATGCA	NM_022629.1	281 bp		-		-3.38	0.997	1.98
	GGAGTCCTGCTCTGGCCTCCT								
CANX	CCAGATGCAGATCTGAAGAC	NM_172008	175 bp	-3.30	1.000	2.01	-3.45	0.999	1.95
	CTGGGTCCTCAATTTCACGT								
MDH1	CAGACAAAGAAGAGGTTGCC	NM_033235.1	206 bp	-3.41	0.999	1.96	-3.27	0.999	2.02
	CGTCAGGCAGTTTGTATTGG								
RPL13	CTTAAATTGGCCACGCAGCT	XR_086310	198 bp	-3.20	0.999	2.05	-3.53	0.997	1.92
	CTTCTCAACGTCTTGCTCTG								
SLC22A5	GAACTCACGAGCCTCGCACGC	NM_019269.1	117 bp	-2.98	1.000	2.16	-3.51	1.000	1.93
	TCGTCGTAGTCCCGCATGCC								
TOP1	GAAGAACGCTATCCAGAAGG	NM_022615	137 bp	-3.45	0.999	1.95	-3.34	0.998	1.99
	GCTTTGGGACTCAGCTTCAT								
YWHAZ	GACGGAAGGTGCTGAGAAA	NM_013011	198 bp	-3.11	0.999	2.10	-3.30	0.999	2.01
	GCAGCAACCTCAGCCAAGT								

^#^Coefficient of determination of the standard curve. *The efficiency was determined by [10^-slope^].

### Western blotting

Homogenates were prepared from frozen tissue aliquots using RIPA buffer (50 mM Tris, 150 mM NaCl, 10% glycerol, 0.1% SDS, 1% Trition X-100, 1 mM EDTA, 0.5% deoxycholate, 1% protease inhibitor mix; pH 7.5). Using the bicinchoninic acid protein assay kit (Interchim, Mantluçon, France) the protein concentrations in the homogenates were determined with BSA as standard. 30 μg protein of each homogenate was separated on 12.5% SDS-PAGE and finally electrotransferred to a nitrocellulosemembrane (Pall, Pensacola, USA). To verify the loading of equal amounts of protein Ponceau S (Carl Roth, Karlsruhe, Germany) staining was used. After incubation of the membranes with blocking solution for 1 hour, the membranes were incubated with the primary antibodies against OCTN2 (polyclonal Anti-Solute carrier family 22 member 5 antibody; Abcam, Cambridge, UK), BBD (monoclonal anti-BBOX1 antibody; Abcam, Cambridge, UK), TMABA-DH (polyclonal anti-TMABA-DH antibody, Abnova, Taipei, Taiwan), and glyceraldehyde-3-phosphate dehydrogenase (GAPDH) (monoclonal anti-GAPDH antibody, Abcam, Cambridge, UK) as a reference protein. The membranes were washed, and then incubated with a horseradish peroxidase-conjugated secondary monoclonal anti-mouse-IgG antibody (Sigma-Aldrich, Steinheim, Germany) for BBD and GAPDH and polyclonal anti-rabbit-IgG antibody (Sigma-Aldrich, Steinheim, Germany) for OCTN2 and TMABA-DH. Afterwards, blots were developed using ECL Select (GE Healthcare, München, Germany). The signal intensities of the specific bands were detected with a Bio-Imaging system (Syngene Cambridge, UK) and quantified using Syngene GeneTools software (nonlinear dynamics).

### Statistics

Statistical analysis of all data was done by one-way ANOVA using the Minitab Statistical Software (Rel. 13.0, State College, PA, USA). Means of the three groups were compared by Fisher’s multiple range test. Means were considered significantly different for P < 0.05. Data presented are shown as means ± SEM.

## Results

### Body weights, feed intake and feed conversion ratio

Initial and final body weights were higher in the obese niacin group (362 ± 10 g; 506 ± 14 g; n = 10) and the obese control group (356 ± 6 g; 501 ± 8 g; n = 10) than in the lean group (270 ± 3 g; 366 ± 5 g; n = 10) (P < 0.05). Daily body weight gains and daily feed intake were higher in the obese niacin group (5.09 ± 0.22 g; 26.6 ± 0.7 g/d; n = 10) and the obese control group (5.12 ± 0.14 g; 25.4 ± 0.6 g/d; n = 10) than in the lean group (3.35 ± 0.10 g; 19.7 ± 0.3 g/d; n = 10) (P < 0.05). Feed conversion ratio was higher in the lean group (5.92 ± 0.13 g feed/g body weight gain; n = 10) than in the obese niacin group (5.27 ± 0.14 g feed/g body weight gain; n = 10) and the obese control group (4.98 ± 0.09 g feed/g body weight gain; n = 10) (P < 0.05). The obese niacin group and the obese control group did not differ with regard to these parameters.

### Concentration of NA and its metabolites (NAM and NUA) in plasma

Rats of the obese niacin group had higher plasma NAM concentrations than those of the obese control group and the lean group (1.2 ± 0.09; 0.6 ± 0.07; 0.5 ± 0.03 μg/ml; n = 10; P < 0.05). The plasma NAM concentration did not differ between the obese control group and the lean group. Plasma concentrations of NA and NUA were below the limit of detection (0.01 μg/ml) in all groups.

### Concentration of carnitine and its precursors in plasma, gastrocnemius muscle and liver

The concentration of total carnitine in plasma and muscle was higher in the lean group than in the two obese groups, but it was higher in the obese niacin group than in the obese control group (P < 0.05; Table 2). Concentration of total carnitine in the liver was also higher in the obese niacin group than in the obese control group (P < 0.05; Table 2), whereas it did not differ between the lean group and the obese control group.

**Table 2:** **Concentrations of total carnitine and its precursors (BB and TML) in plasma, liver and gastrocnemius muscle of lean rats (Lean), obese Zucker rats fed a control diet (Obese Control) or obese Zucker rats fed a diet supplemented with 780 mg niacin/kg diet (Obese Niacin) for 4 wk**^**1**^

	**Lean**	**Obese control**	**Obese niacin**
*Plasma (μmol/l)*			
Total Carnitine	62.4 ± 2.1^a^	39.5 ± 1.1^c^	48.4 ± 2.9^b^
γ-Butyrobetaine (BB)	0.75 ± 0.04^a^	0.36 ± 0.04^b^	0.42 ± 0.05^b^
Trimethyllysine (TML)	0.89 ± 0.03^a^	0.64 ± 0.02^b^	0.67 ± 0.03^b^
*Skeletal muscle (nmol/g)*			
Total Carnitine	730 ± 26^a^	602 ± 28^b^	681 ± 28^a^
γ-Butyrobetaine (BB)	10.9 ± 0.5^a^	8.42 ± 0.40^ab^	7.31 ± 0.65^b^
Trimethyllysine (TML)	73.7 ± 11.5	58.7 ± 10.5	58.8 ± 3.8
*Liver (nmol/g)*			
Total Carnitine	289 ± 10^b^	298 ± 7^b^	334 ± 12^a^
γ-Butyrobetaine (BB)	3.32 ± 0.35^a^	2.12 ± 0.13^b^	1.93 ± 0.17^b^
Trimethyllysine (TML)	16.5 ± 1.6	13.5 ± 0.7	14.1 ± 0.8

^1^Data are expressed as means ± SEM. n = 10 rats/group. ^a,b^Values with different superscript letters differ, P < 0.05.

Concentrations of the carnitine precursor BB in plasma, muscle and liver were higher in the lean group than in the obese control group and the obese niacin group (P < 0.05; Table 2), but did not differ between the two obese groups. The concentration of the carnitine precursor TML in plasma was higher in the lean group than in the two obese groups (P < 0.05; Table 2), whereas it was not different between these groups in liver and skeletal muscle.

### Relative mRNA concentrations of PPAR target genes involved in lipid metabolism of liver and gastrocnemius muscle

To investigate whether niacin causes an activation of PPARα and/or PPARδ in muscle and liver, we determined the mRNA concentrations of classical PPAR target genes involved in fatty acid oxidation in these tissues. Relative mRNA concentrations of acyl-CoA-oxidase 1 (ACOX1), short-chain acyl-CoA-dehydrogenase (ACADS), medium-chain acyl-CoA-dehydrogenase (ACADM) and long-chain acyl-CoA-dehydrogenase (ACADL), which are involved in peroxisomal and/or mitochondrial β-oxidation, in liver and skeletal muscle were higher in the obese niacin group than in the obese control group and the lean group (P < 0.05; Table 3).

**Table 3:** **Relative mRNA concentrations of PPARα and PPARδ target genes involved in β-oxidation (ACOX1, SCAD, MCAD, LCAD) and genes involved in carnitine uptake (OCTN2) and biosynthesis (BBD, TMABA-DH) in liver and gastrocnemius muscle of lean rats (Lean), obese Zucker rats fed a control diet (Obese Control) or obese Zucker rats fed a diet supplemented with 780 mg niacin/kg diet (Obese Niacin) for 4 wk**^**1**^

	**Lean**	**Obese control**	**Obese niacin**
	**Relative mRNA concentration (fold of obese control = 1.00)**		
*Skeletal muscle*			
ACOX1	1.49 ± 0.29^ab^	1.00 ± 0.21^b^	1.89 ± 0.36^a^
SCAD	1.68 ± 0.46^ab^	1.00 ± 0.40^b^	2.69 ± 0.70^a^
MCAD	1.40 ± 0.30^ab^	1.00 ± 0.34^b^	2.24 ± 0.47^a^
LCAD	1.43 ± 0.46^ab^	1.00 ± 0.25^b^	3.78 ± 1.32^a^
SLC22A5	1.25 ± 0.31^ab^	1.00 ± 0.18^b^	1.93 ± 0.42^a^
*Liver*			
ACOX1	0.92 ± 0.14^b^	1.00 ± 0.12^b^	1.42 ± 0.07^a^
SCAD	0.91 ± 0.09^b^	1.00 ± 0.14^b^	1.57 ± 0.08^a^
MCAD	1.19 ± 0.13^b^	1.00 ± 0.14^b^	1.76 ± 0.22^a^
LCAD	0.99 ± 0.17^b^	1.00 ± 0.14^b^	1.57 ± 0.08^a^
SLC22A5	0.78 ± 0.06^b^	1.00 ± 0.07^b^	1.42 ± 0.12^a^
BBOX1	0.76 ± 0.08^b^	1.00 ± 0.08^b^	1.54 ± 0.06^a^
ALDH9	1.00 ± 0.08^b^	1.00 ± 0.08^b^	1.40 ± 0.14^a^

^1^Data are expressed as means ± SEM. n = 10 rats/group. ^a,b^Values with different superscript letters differ, P < 0.05.

### Relative mRNA concentrations of genes involved in carnitine uptake in liver and gastrocnemius muscle and carnitine synthesis in the liver

To study whether niacin influences the expression of genes involved in carnitine uptake in liver and muscle and hepatic carnitine synthesis, we evaluated the mRNA concentrations of SLC22A5, BBOX1 and ALDH9 in liver and muscle, respectively. BBOX1 and ALDH9 were considered only in the liver, because the liver is the major organ responsible for carnitine biosynthesis. In the liver, the mRNA concentrations of SLC22A5, BBOX1 and ALDH9 were higher in the obese niacin group than in the obese control group and the lean group (P < 0.05; Table 3), but they were not different between the obese control group and the lean group. The mRNA concentration of SLC22A5 in gastrocnemius muscle was also higher in the obese niacin group than in the obese control group (Table 3), but did not differ between the obese niacin group and the lean group.

### Relative protein concentrations of genes involved in carnitine uptake in liver and gastrocnemius muscle and carnitine synthesis in the liver

**Figure 1:**
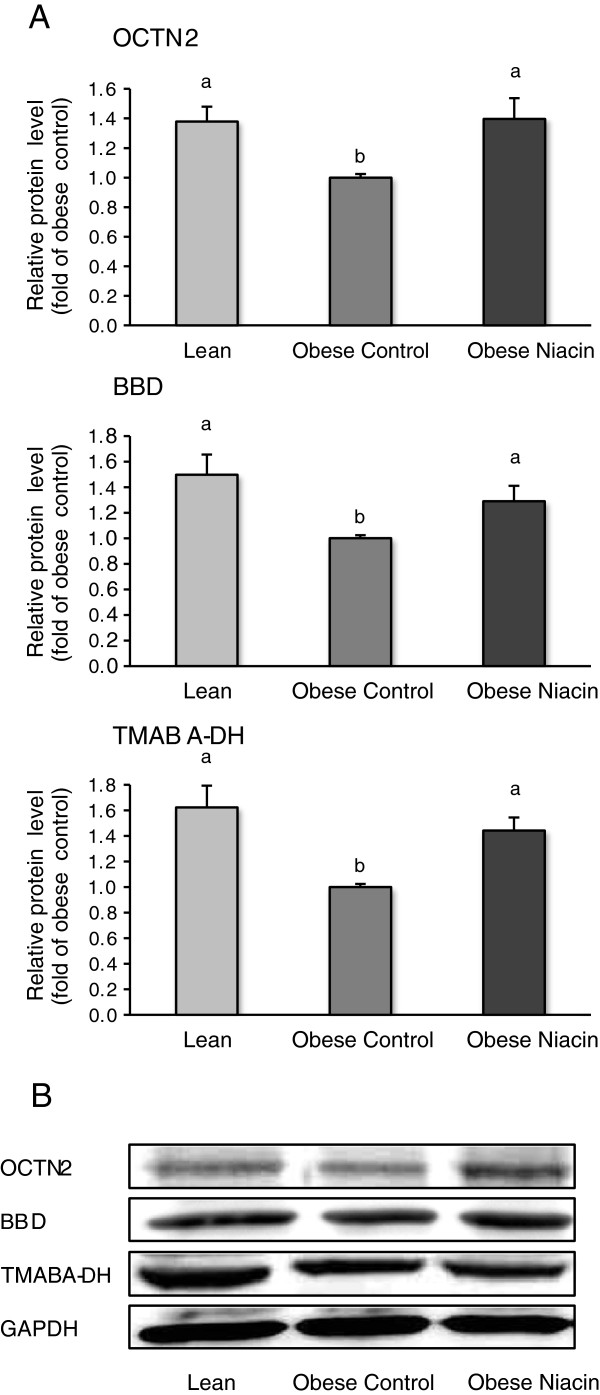
**Relative protein levels of OCTN2**, **BBD and TMABA-DH in liver of lean rats (Lean), obese Zucker rats fed a control diet (Obese Control) or obese Zucker rats fed a diet supplemented with 780 mg niacin/kg diet (Obese Niacin) for 4 wk. (A)** Bars represent means ± SEM, n = 10/group. Means without a common letter differ, P < 0.05. **(B)** Representative immunoblots specific to OCTN2, BBD, TMABA-DH and GAPDH as internal control are shown for one animal per group; immunoblots for the other animals revealed similar results.

**Figure 2:**
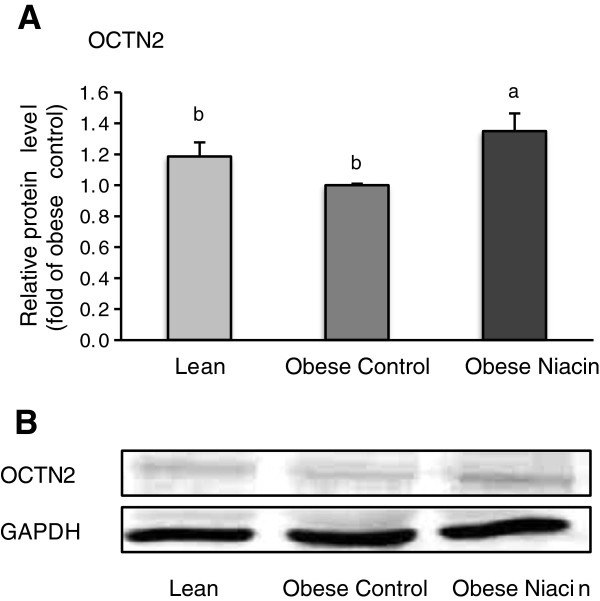
**Relative protein level of OCTN2 in *M. gastrocnemius *of lean rats (Lean), obese Zucker rats fed a control diet (Obese Control) or obese Zucker rats fed a diet supplemented with 780 mg niacin/kg diet (Obese Niacin) for 4 wk. (A)** Bars represent means ± SEM, n = 10/group. Means without a common letter differ, P < 0.05. **(B)** Representative immunoblots specific to OCTN2 and GAPDH as internal control are shown for one animal per group; immunoblots for the other animals revealed similar results.

Relative protein concentrations of OCTN2, BBD, and TMABA-DH in the liver were lower in the obese control group than in the obese niacin group and the lean group (P < 0.05; Figure 1), but they were not different between the obese niacin group and the lean group. Relative protein concentration of OCTN2 in muscle was higher in the obese niacin group than in the obese control group and the lean group (P < 0.05; Figure 2).

## Discussion

In the present study, we tested the hypothesis that niacin administration to obese Zucker rats is able to improve the carnitine status of obese Zucker rats through PPAR-stimulated expression of genes involved in carnitine uptake and biosynthesis. As expected, administration of 780 mg niacin/kg diet for 4 wk to obese Zucker rats resulted in an about 2-fold elevation of plasma NAM levels compared to obese control rats, which received a physiological niacin dose (30 mg/kg diet) sufficient to cover their niacin requirement. This elevation of plasma NAM levels was in the range of that reported in other studies, in which a similar niacin dose was fed to rats [25,28]. The finding that nicotinic acid and NUA were not detectable was also not unexpected, because nicotinic acid from the diet is rapidly converted to NAD and NAM in the intestine and liver, from which NAM is released into the blood stream [29]. Nicotinic acid and NUA can be found in the systemic blood at significant levels only when nicotinic acid is administered either i.v. or as a fast release preparation, because nicotinic acid is then activated by combination with coenzyme A (CoA) to form nicotinyl-CoA, which is then conjugated with glycine to NUA [30]. A key finding of the present study was that niacin administration in pharmacological doses results in enhanced carnitine concentrations in plasma, skeletal muscle and liver and improves the diminished carnitine status of obese Zucker rats indicating that niacin increases carnitine uptake into tissues and hepatic carnitine synthesis. Although it has been proposed that the carnitine precursors TML and BB are rate-limiting for carnitine biosynthesis [31], we found no difference in the concentrations of carnitine precursors between the two obese groups, despite elevated carnitine concentrations in plasma and liver in the obese niacin group. However, the observation from the present study that mRNA and protein levels of OCTN2, BBD and TMABA-DH were significantly increased in the liver and skeletal muscle of the obese niacin group compared to the obese control group rather suggests that the rate of carnitine biosynthesis and uptake were elevated through stimulating expression of genes involved in carnitine homeostasis. Noteworthy, BB is also a good substrate for OCTN2 [32,33] and the liver has a high capacity to convert BB into carnitine [34]. Thus, it is likely that the niacin-induced increase in the expression of OCTN2 in the liver also contributed to an elevated carnitine biosynthesis rate in the liver.

In view of recent indications that niacin causes activation of PPARα and/or PPARδ in tissues of rats [19], and activation of both of them is known to stimulate carnitine uptake and biosynthesis [12,14-18], we studied whether niacin resulted in the activation of PPARα and PPARδ in the liver and skeletal muscle of obese Zucker rats. We observed that several PPARα and PPARδ target genes, such as ACOX1, ACADS, ACADM, ACADL, encoding enzymes of fatty acid β-oxidation were elevated in both, liver and skeletal muscle, of the obese niacin group compared to the obese control group indicating that PPARα and/or PPARδ was indeed activated by niacin administration. Regarding that mRNA and protein levels of OCTN2, BBD and TMABA-DH, which have been identified either as direct PPARα target genes or to be regulated by PPARδ [12,14-16], were increased in the liver and skeletal muscle of the obese niacin group compared to the obese control group suggests that activation of PPARα and/or PPARδ is responsible for this effect. Since the selected genes encoding enzymes of fatty acid β-oxidation but also other genes involved in fatty acid β-oxidation are target genes of both, PPARα and PPARδ, it is not possible to differentiate whether niacin activates either PPARα or PPARδ or both of them. However, recent observations from whole-genome transcriptional profiling in PPARα knockout and PPARδ knockout mice suggested that the key regulator of fatty acid oxidation genes in the liver is PPARα [35], whereas PPARδ, the predominant PPAR isoform in skeletal muscle, plays this role in skeletal muscle. It is therefore possible that the effect of niacin on OCTN2 expression in skeletal muscle is mediated by PPARδ activation and that on OCTN2, BBD and TMABA-DH expression in the liver by PPARα activation.

The mechanism underlying activation of PPARα and/or PPARδ by niacin remains to be resolved but it may involve an elevation of circulating levels of NEFA, which serve as PPAR ligands. Considering the well-known antilipolytic effect of niacin, an elevation of plasma NEFA levels seems paradox. However, the rapid decrease of plasma NEFA concentrations in response to acute administration of niacin is followed by a marked rebound of plasma NEFA levels to even above pre-treatment levels [36,37], which may be responsible for an increased binding to and activation of PPARs in tissues. The rebound phenomenon has been explained by the decreasing antilipolytic effect of the administrated niacin and the parallel stimulation of lipolysis by lipolytic hormones, like epinephrine and corticosterone, which are found at increased levels in plasma following niacin administration [7,38]. Nevertheless, based on the present data it still remains speculative if the effect of niacin on carnitine homeostasis is mediated by activation of PPARs. Definitive proof can be provided only by investigating the effect of niacin in PPARα/PPARδ double-knockout mice in future studies.

Since there is no evidence in the literature that niacin supplementation stimulates protein catabolism in skeletal muscle, we exclude the possibility that niacin caused the improvement of carnitine status through an enhanced release of the carnitine precursor TML, which is released upon protein degradation, from skeletal muscle. In line with this assumption, we found no difference in the TML concentrations in plasma, liver and skeletal muscle, the main source of TML, between the obese control and the obese niacin group.

## Conclusion

Our study shows for the first time that niacin administration is able to improve the diminished carnitine status of obese Zucker rats, an established genetic model of human obesity. The herein reported effect of niacin on the carnitine status is supposed to be beneficial in obese subjects, because it is well-known that obesity causes an impairment of carnitine status, which was evident also in the obese Zucker rats. An impaired carnitine status is generally detrimental for the metabolic situation in obese subjects, which is characterized by a chronic excess of metabolic substrates, because it contributes not only to reduced fatty acid oxidation capacity and to mitochondrial dysfunction but also to insulin resistance [20]. Thus, normalizing an impaired carnitine status is a key for improving the metabolic situation during obesity as evidenced from the observation that oral carnitine supplementation overcomes not only mitochondrial dysfunction but also improves glucose tolerance in different rodent models of obesity [20]. One important factor being causative for the impaired carnitine status during obesity is likely a disturbed PPARα function [21] leading to a decreased capacity of the liver to synthesize and take up carnitine, because PPARα is a critical transcriptional regulator of genes involved in carnitine homeostasis [12,15-18]. This is also evident from the finding that PPARα knockout mice have markedly decreased levels of carnitine in tissues and plasma [39]. Thus, activation of PPARα in tissues by niacin is likely responsible for the increased carnitine concentration in the liver of obese Zucker rats. In agreement with this, we have recently demonstrated that regular endurance exercise, which also causes activation of PPARs in tissues due to an elevated release of NEFA from adipose tissue, is able to restore the reduced carnitine levels in high-fat diet-induced obese mice to those levels found in non-obese sedentary mice due to activation of PPARs and PPAR co-activators in the liver and enhancing the mRNA and protein levels of genes involved in carnitine uptake and synthesis [22].

## Competing interests

The authors declare that they have no competing interests.

## Authors’ contributions

AC carried out the molecular biological analyses, performed statistical analysis, and drafted the manuscript. RR participated in the design of the study, and helped to draft the manuscript. EM performed analysis of nicotinic acid and its metabolites. KE conceived of the study, and participated in its design and coordination and helped to draft the manuscript. All authors read and approved the final manuscript.

## Pre-publication history

The pre-publication history for this paper can be accessed here:

http://www.biomedcentral.com/2050-6511/15/37/prepub
